# The accuracy of D-dimer in the diagnosis of periprosthetic infections: a systematic review and meta-analysis

**DOI:** 10.1186/s13018-022-03001-y

**Published:** 2022-02-16

**Authors:** Renwei Wang, Hui Zhang, Peng Ding, Qiang Jiao

**Affiliations:** 1 Department of Orthopedic, Second Hospital of Shanxi Medical University, Taiyuan, 030000 Shanxi Province China; 2 Second Hospital of Shanxi Medical University, Taiyuan, 030000 Shanxi Province China

**Keywords:** D-dimer, Periprosthetic infection, Diagnostic meta-analysis

## Abstract

**Background:**

Periprosthetic joint infection (PJI) is a devastating complication after total hip arthroplasty (THA) or total knee arthroplasty (TKA). It is scarce and contradicting evidence supporting the use of serum D-dimer to diagnose PJI in revision THA and TKA. This systematic review and meta-analysis aimed to investigate the accuracy of D-dimer in the diagnosis of periprosthetic infections.

**Methods:**

The PubMed, Embase, Web of Science were systematically searched from the inception dates to August 15, 2020. We included all diagnostic studies of D-dimer in the diagnosis of periprosthetic infections. The literature's quality was evaluated by the QUADAS-2 tool, and Stata16 and Revman5.3 software carried out the meta-analysis.

**Results:**

Of 115 citations identified by the search strategy, 10 studies (comprising 1756 participants) met the inclusion criteria**.** The literature quality assessment results show that most of the literature is low-risk bias literature. The combined sensitivity of D-dimer in diagnosing periprosthetic infections was 0.81 (95% confidence interval [CI] 0.71–0.88), combined specificity was 0.74 (95% CI 0.61–0.84), combined positive likelihood ratio was 3.1 (95% CI 2.0–5.0), combined negative likelihood ratio 0.26 (95% CI 0.16–0.41), combined diagnosis odds ratio 12 (95% CI 5–27), area under the Summary Receiver Operator Characteristic Curve (SROC) is 0.85 (95% CI 0.81–0.88). The data are statistically significant.

**Conclusion:**

D-dimer has a high diagnostic value in diagnosing PJI and has clinical significance in diagnosing periprosthetic infection. In addition, there are relatively few studies on the threshold of D-dimer, different sampling types, different laboratory detection methods, and different races, so more prospective trials with large samples, multi-centers, and scientific design should be carried out in the future.

**Supplementary Information:**

The online version contains supplementary material available at 10.1186/s13018-022-03001-y.

## Introduction

The PJI has become one of the most frequent and devastating complications of lower-extremity total joint arthroplasty, accounting for 25% of TKA failures and 16% of THA failures [1, 2]. The cost of PJI is estimated to be over $5.66 million and is expected to 2020 will exceed $162 million [3]. The incidence of PJI ranged from 0.5% to 2.0% due to atypical symptoms in many PJI patients [4]. Making a timely and accurate diagnosis of PJI has remained a challenge for orthopaedic surgeons [5].

The Musculoskeletal Infection Society (MSIS) published the diagnostic criteria for PJI in 2011 [6], which were later improved and modified at the 2013 International Consensus Meeting (ICM) [7]. MSIS and ICM criteria were developed based on clinical presentation, pathogen culture results, blood tests, synovial fluid examination, and histological analysis for the global diagnosis of PJI. The sensitivity of ICM and MSIS criteria was 86.9% and 79.3% [8]. The D-dimers are fibrin degradation products formed by fibrin clot fibrinolysis, reflecting the state of blood coagulation, and are increased in systemic or local infections, thrombosis, and neoplastic diseases.[9–11]. Shahi et al.[12] were first found that serum dimer levels performed well in determining PJI with a sensitivity of 89% and a specificity of 93%. Li et al. [13] speculated that D-dimer in patients with PJI might be a biomarker for late diagnosis. In 2018, Parvizi et al.[8] presented new evidence regarding the diagnosis of PJI based on evidence-based medicine that included D-dimer levels as a secondary criterion. However, some studies have found that D-dimer is limited in diagnosing periprosthetic infections. Pannu et al. [14] believed that D-dimer has poor accuracy (61%) and low specificity (32.3%) in identifying PJI and aseptic loosening. Xiong et al. [15] concluded that the diagnostic efficiency of D-dimer was not superior to that of C-reactive protein (CRP) and erythrocyte sedimentation rate (ESR). The correlation Meta-analysis by Cheng Li et al. [16] showed that D-dimer’s diagnostic sensitivity and specificity for PJI was low, and the heterogeneity of the sensitivity and specificity obtained from the included studies was significant. To analyze the reasons for the heterogeneity, performed a subgroup analysis of serum and plasma D-dimer. They concluded that serum D-dimer had a better diagnostic value for PJI than plasma D-dimer. There is no agreement on the findings of other similar studies. Therefore, the diagnostic value of D-dimer is still controversial.

To the best of our knowledge, there is a lack of high-level evidence for the accuracy of D-dimer in the diagnosis of PJI. The purpose of this systematic review and meta-analysis was to assess the diagnostic value of D-dimer in the diagnosis of PJI, and if there was heterogeneity in the results, to search for potential sources of heterogeneity by univariate regression analysis, including blood sample type, infection at other sites, diagnostic criteria, prosthesis type, study type, country, and threshold.

## Materials and methods

### Study design

We did a systematic review and meta-analysis following the PRISMA (Preferred Reporting Items for Systematic reviews and meta-Analyses) guidelines and used a predetermined protocol [17].

### Search strategy

The PubMed, Embase, Web of Science were systematically searched from the inception dates to August 15, 2020. Two researchers conducted the literature search, and the search process was independent and double-blind. The systematic evaluation included only diagnostic studies of D-dimer in the diagnosis of periprosthetic infections.Taking the PubMed database as an example, the English search strategy was: #1 D-dimer * OR D-dimer fibrin * OR D-dimer fragments * OR fibrin fragment D-dimer * OR fibrin fragment DD *#2periprosthetic joint infection * OR prosthesis-related infections #3 #1 AND #2.

### Inclusion criteria and exclusion criteria

Inclusion criteria: (1) D-dimer as an indicator for the diagnosis of PJI [8]; (2) direct or indirect true positive (TP), false positive (FP), true negative (TN), false negative (FN) data; (3) clear gold standard such as MSIS [6] or ICM [7] criteria to compare the diagnostic D-dimer accuracy. (4) The article is a diagnostic pilot study. Exclusion criteria: (1) studies assessing the diagnostic value of blood or synovial fluid biomarkers other than D-dimer were excluded. (2) Animal studies; (3) literature was a review or meta-analysis; (4) study sample size was less than 10.

### Study selection

The literature screening was done by two independent researchers, who initially selected the literature that met the inclusion criteria by reading the title and abstract of the literature, the full text of the initially included literature was read, and those that did not meet the requirements were excluded according to the exclusion criteria, and the included literature was further reviewed and evaluated.

### Data extraction

Data extraction was performed independently by two researchers by reading the full text of the included literature and extracting the following literature information. (1) basic information of the literature, including authors, year of publication, study type, country, and sample size; (2) general patient information, including age, gender, and baseline status; (3) diagnostic gold standard, sampling type, D-dimer threshold, and other evaluation indicators. (4) Detailed true-positive, false-positive, true-negative, and false-negative data used to construct the 2 × 2 table were recorded. See Table 1. The extracted information was cross-checked, and in case of disagreement a third investigator was involved in the determination.

**Table 1:** Extracted data used to construct 2 × 2 tables

Author	Year	TP	FP	FN	TN	Total
Wang [21]	2020	34	15	17	91	157
Qin [22]	2019	51	17	4	50	122
Pannu [14]	2020	47	42	2	20	111
Hu [23]	2020	35	4	5	33	77
Shahi [12]	2017	51	10	6	128	195
Fu [19]	2019	10	6	5	9	30
Li [13]	2019	60	165	35	305	565
Huang [20]	2019	22	14	9	56	101
Xiong [15]	2019	21	11	5	43	80
Xu [24]	2019	88	93	41	96	318

### Quality evaluation

The QUADAS-2 tool was used to evaluate the included literature. The QUADAS-2 tool consists of 4 domains: Participant selection, Index test, Reference standard, Flow, and timing. Each part is assessed based on the risk of bias, and the first three domains are also evaluated based on applicability. Compared to QUADAS has more accurate bias ratings and relevance for diagnostic studies [18].

### Statistical analysis

The quality of the included literature was evaluated using the QUADAS-2 tool in Review Manager 5.3 analysis software, and the Spearman rank correlation coefficient was calculated using Stata 16 software to assess threshold effects and combine effect sizes. Spearman's rank correlation coefficient was significantly positive (or p-value less than 0.05), or SROC the curve showed a "shoulder-arm" point distribution, suggesting a threshold effect and heterogeneity. Heterogeneity was tested using the test; if *I*^2^ < 50%, then heterogeneity among studies was considered small and fixed-effects model was used; if *I*^2^ > 50%, then heterogeneity among studies was deemed to be a large and random-effects model was used, and regression and subgroup analysis were performed to determine the source of heterogeneity. The variables that we believe may influence heterogeneity are the type of study design, the threshold used for the study, the number of cases, the diagnostic gold index, the type of sample, and the country or region in which the literature was published. According to the combined effect of the corresponding model, the merging sensitivity, merging specificity, merging positive likelihood ratio, combining negative likelihood ratio and combining diagnostic ratio were obtained. After merging, the SROC curve was obtained, and the area under the curve (AUC) was calculated AUC areas ≥ 0.50, 0.75, 0.93, and 0.97 were defined as fair, good, better, and excellent, respectively.

## Results

### Literature search results

**Fig. 1:**
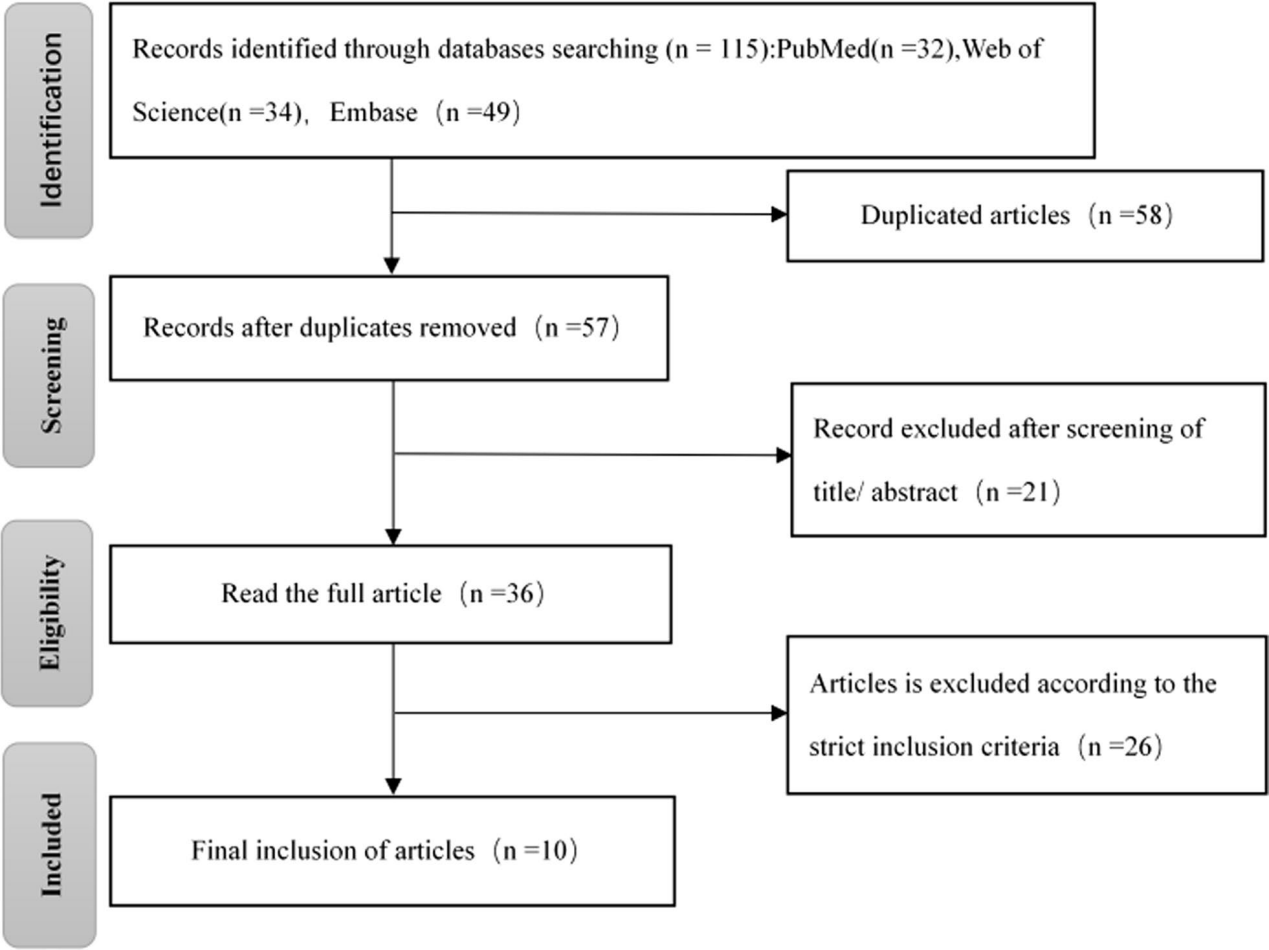
Literature search and selection strategy

The preliminary search obtained 115 publications. Fifty-eight repeated articles were excluded. Thirty-six articles were initially included after reading the titles and abstracts. After further reading of the full text of the literature, 10 articles, all in English, with a total of 1756 subjects, were finally included. The literature search and selection strategy are shown in Fig. 1.

### Basic characteristics of the included literature

Basic information of the included literature (including author, publication, study type, country, and sample size), general patient information, including age, sex, and baseline status, diagnostic gold standard, sampling type, D-dimer threshold, typology of infection, whether the inflammatory disease were excluded, whether a control group was set up, and evaluation indicators such as prosthesis type were extracted. However, unfortunately, the prosthesis material was not mentioned in the original literature. Of the types of infection, only two are chronic, the rest of the literature does not mention the type of infection. General patient information is shown in Table 2.

**Table 2:** Baseline characteristics of a meta-analysis study of D-dimer in the diagnosis of periprosthetic infection

Author	Year	Country	Type of research	Sample size	Case group/control group	Average age	Male/female	Gold Standard	Sampling type	Threshold	Whether to exclude inflammatory diseases	Whether to set a control group	Prosthesis type	Typology of infection
Wang [21]	2020	China	Retrospective	157	51/106	64.6	70/87	MSIS	Serum	1220 ng/ml	Yes	Yes	NA	Chronic
Qin [22]	2019	China	Forward-looking	122	55/67	65.9	53/69	ICM	Serum	1170 ng/ml	Yes	Yes	NA	Chronic
Pannu [14]	2020	USA	Retrospective	111	89/22	70	62/49	ICM	Serum	850 ng/ml	No	Yes	NA	NA
Hu [23]	2020	China	Retrospective	77	40/37	60.8	37/40	MSIS	Serum	955 ng/ml	No	Yes	NA	NA
Shahi [12]	2017	USA	Forward-looking	195	86/109	59.7	101/94	ICM	Serum	850 ng/ml	No	Yes	NA	NA
Fu [19]	2019	China	Forward-looking	30	15/15	65.6	9/21	MSIS	Plasma	850 ng/ml	Yes	Yes	NA	NA
Li [13]	2019	China	Retrospective	565	95/470	NA	248/317	ICM	Plasma	1250 ng/ml	No	Yes	NA	NA
Huang [20]	2019	China	Retrospective	101	31/70	64.9	NA	ICM	Serum	850 ng/ml	Yes	Yes	NA	NA
Xiong [15]	2019	China	Forwardlooking	80	26/54	65.4	54/26	MSIS	Serum	756 ng/ml	Yes	Yes	NA	NA
Xu [24]	2019	China	Retrospective	318	129/189	NA	NA	ICM	Plasma	1020 ng/ml FEU	Yes	Yes	NA	NA

The control group was the aseptic loosening group. NA = not applicable

### Quality evaluation

**Fig. 2:**
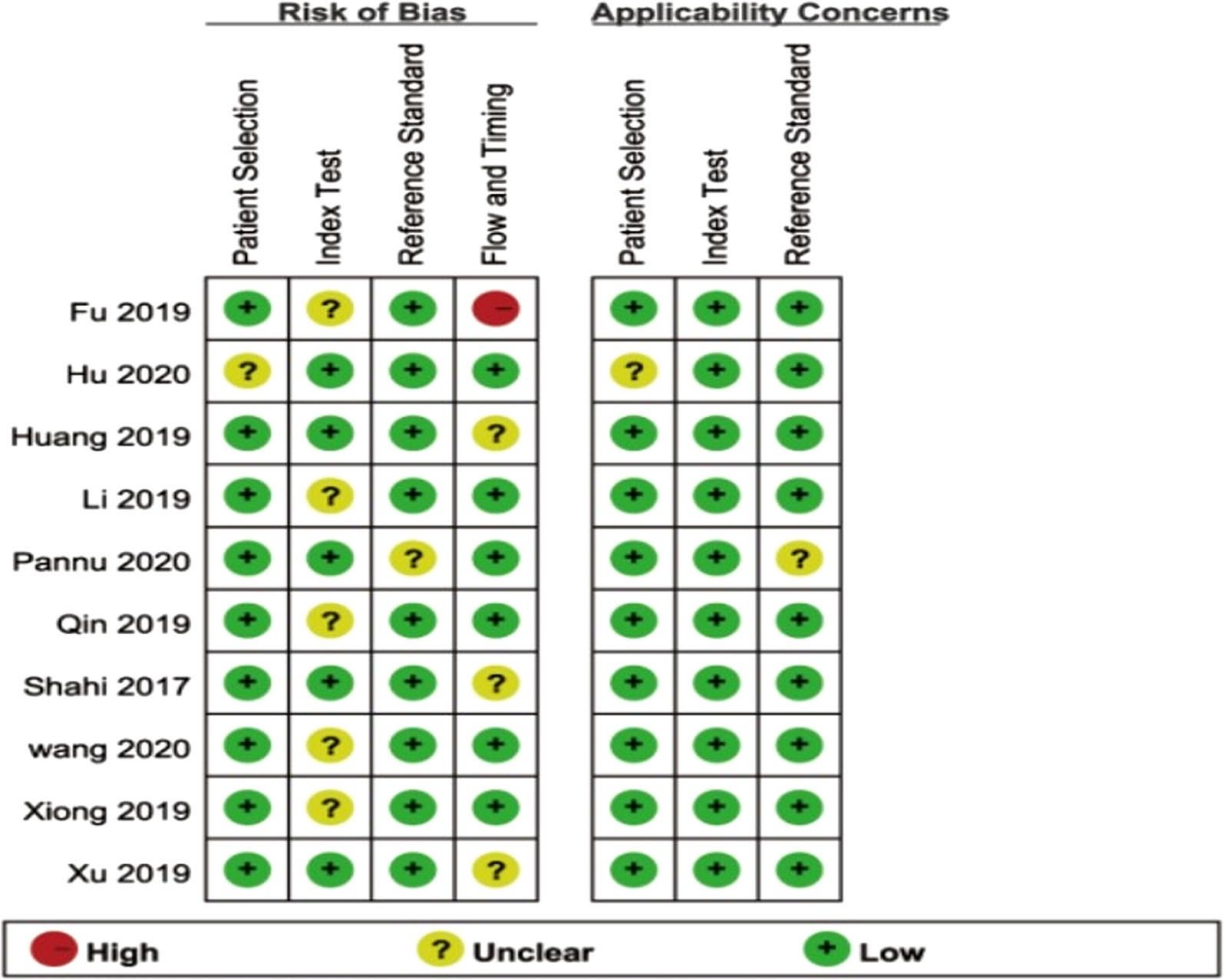
D-dimer diagnostic test quality evaluation diagram in the diagnosis of prosthetic infection

**Fig. 3:**
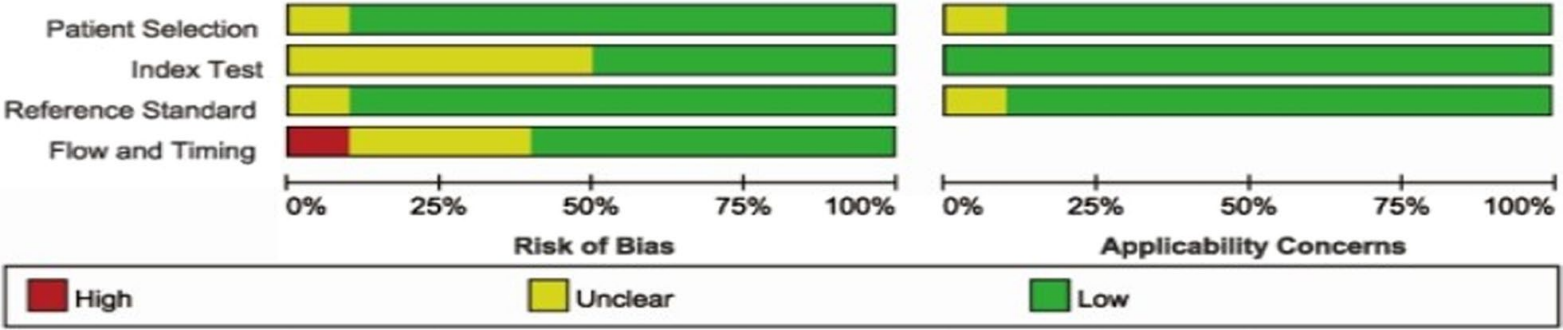
The overall quality evaluation diagram of d-dimer in the diagnosis of infection around the prosthesis

The inclusion of the data in Revman 5.3 for analysis leads to Figs. 2 and 3. Figures 2 and 3 show the quality assessment of the included literature using the QUADAS-2 tool. As can be seen from the figure, most of the literature falls into the low-risk bias literature.

### Threshold and accuracy of D-dimer diagnosis of PJI

**Fig. 4:**
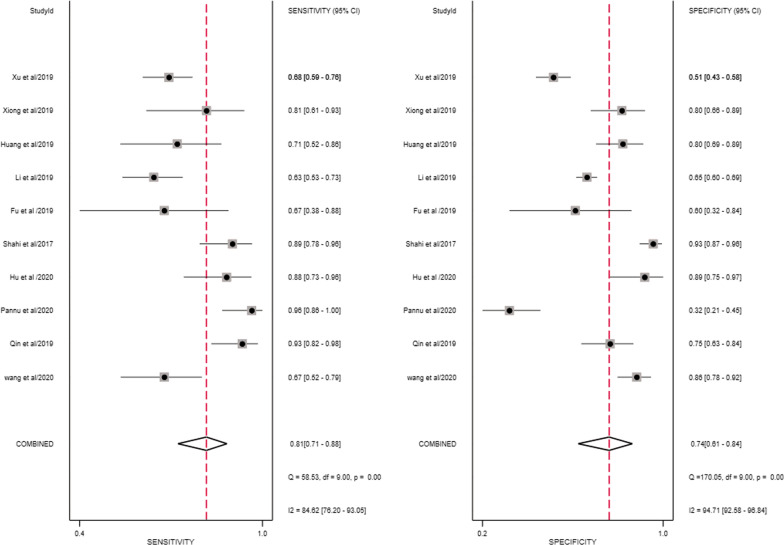
The combined sensitivity and specificity forest plot of d-dimer in the diagnosis of PJI

**Fig. 5:**
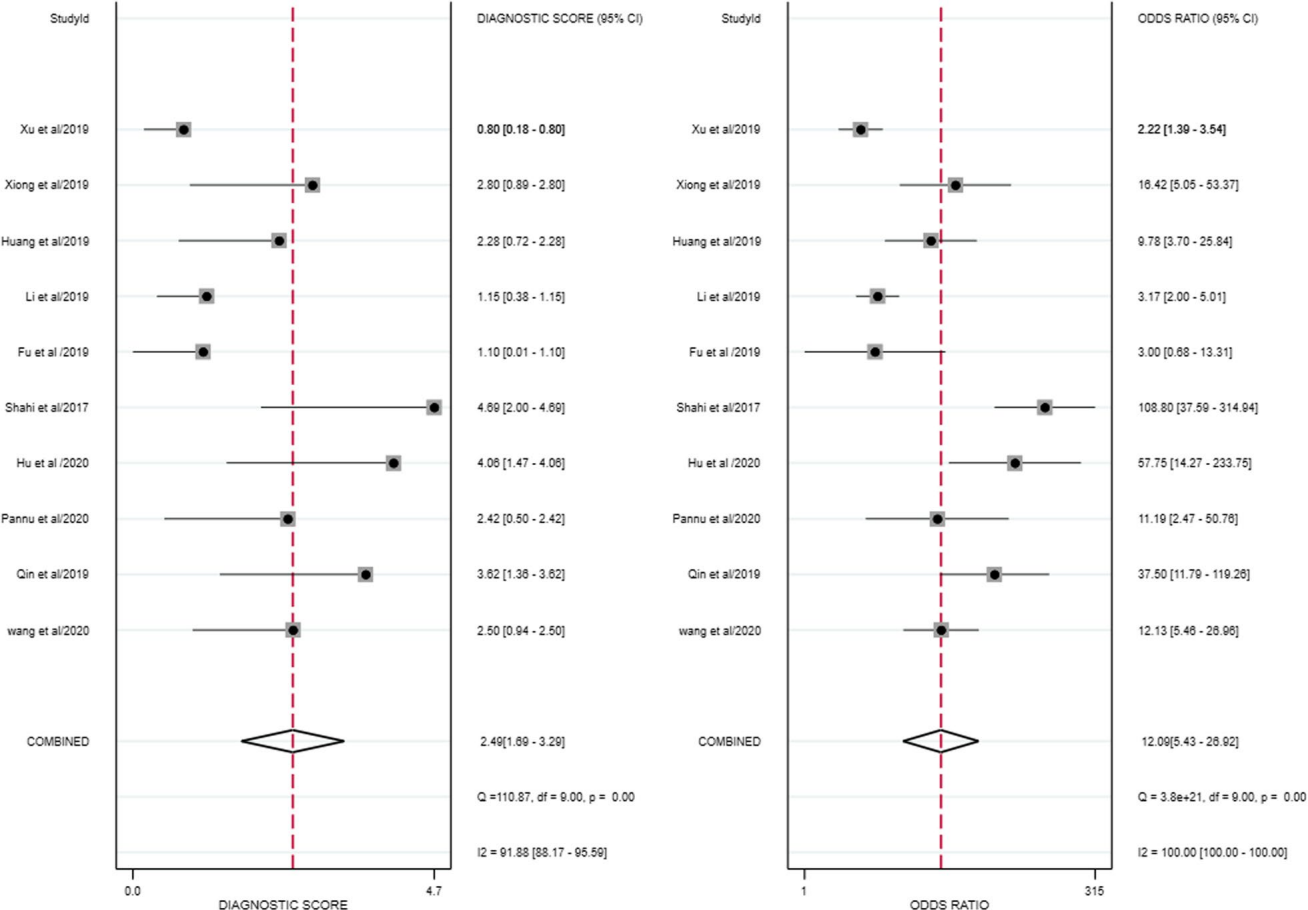
Forest plot of combined diagnostic score and diagnostic odds ratio of d-dimer in the diagnosis of PJI

**Fig. 6:**
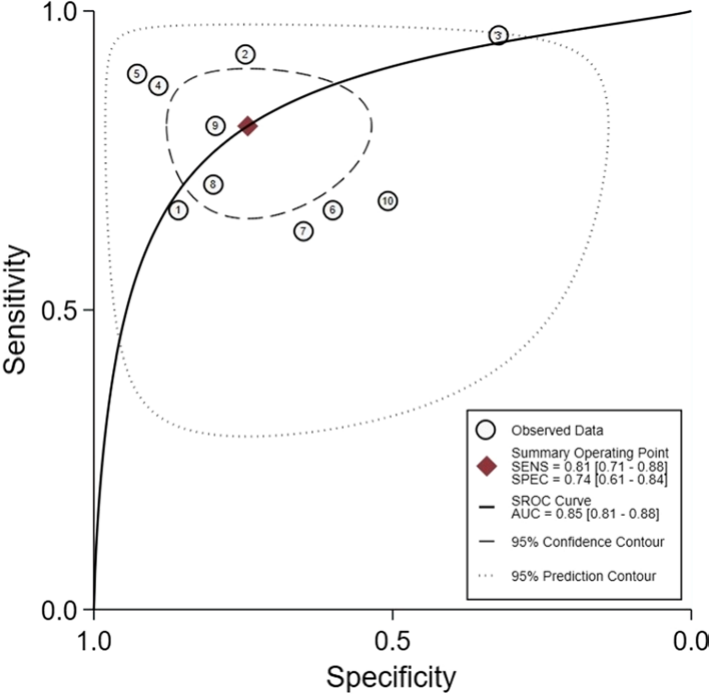
SROC curve of d-dimer diagnosis PJI included in the study

For the thresholds, four studies used a common threshold (850 ng/ml) [12, 14, 19, 20] and the remaining six studies used different threshold [13, 15, 21–24]. The Spearman's correlation coefficient for D-dimer was − 0.17. Since the number of studies in this paper was less than 30, the table was checked to obtain a p-value of 0.7, greater than 0.05, indicating that a threshold effect did not cause the heterogeneity. Similarly, the SROC curve did not show a "shoulder-arm" point distribution, indicating that the threshold effect was insignificant and did not cause heterogeneity. The forest plot showed that the combined sensitivity and specificity of D-dimer for the diagnosis of PJI were 0.81 (95% CI 0.71–0.88), and the combined specificity was 0.74 (95% CI 0.61–0.84) (Fig. 4). The values of I^2^ in the combined sensitivity and specificity forest plot of the corresponding D-dimer for the diagnosis of PJI were 84.62 (95% CI 76.20–93.05) and 94.71 (95% CI 92.58–96.84). This shows that there is a large heterogeneity among the studies. The combined diagnostic score and diagnostic advantage ratio were 2.49 (95% CI 1.69–3.29) and 12.09 (95% CI 5.43–26.92) (Fig. 5).The area under the SROC curve was 0.85 [95% CI 0.81–0.88] (Fig. 6).

### Assessment of clinical utility

**Fig. 7:**
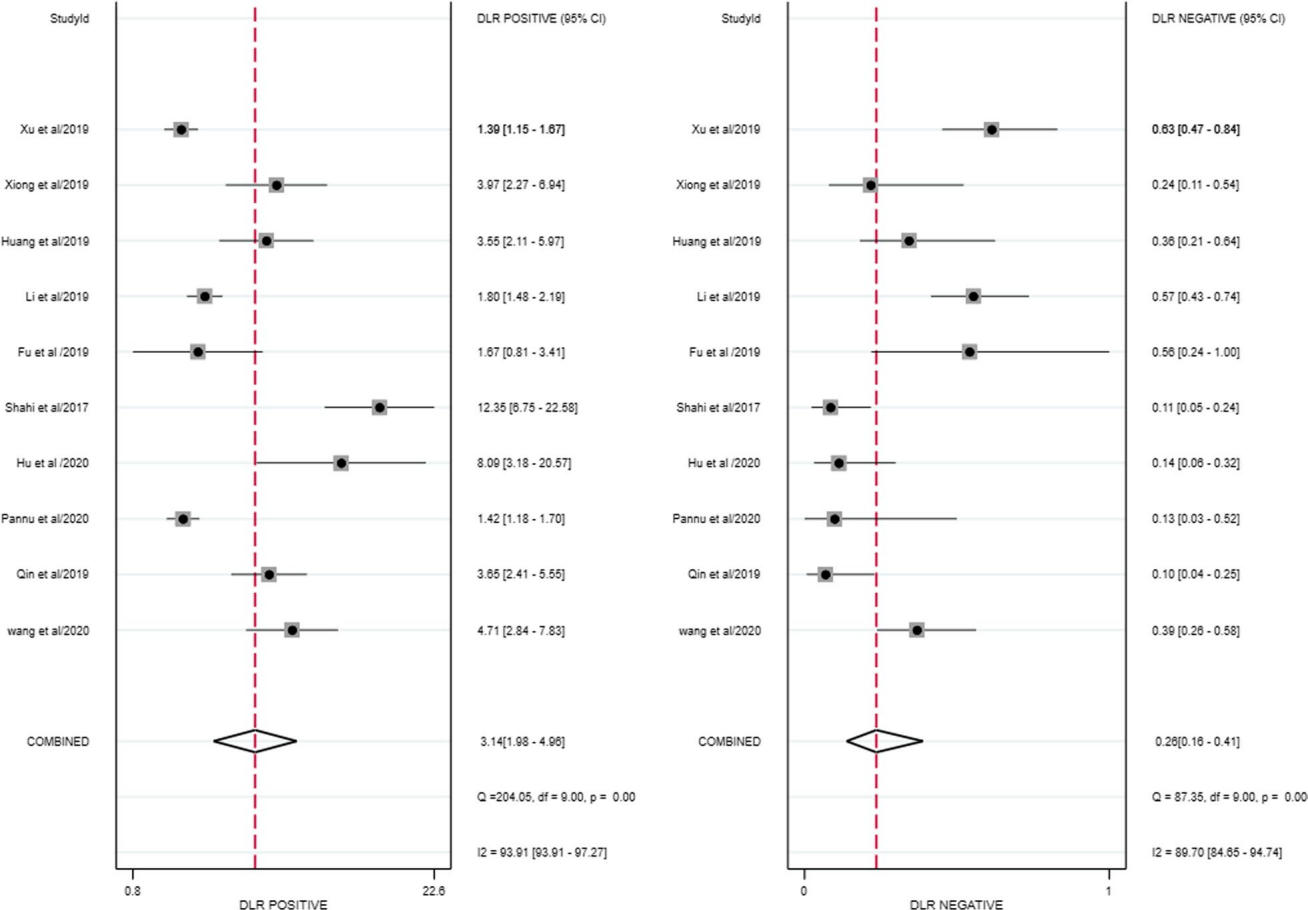
D-dimer diagnosis PJI combined with positive likelihood ratio and negative likelihood ratio forest plot

The combined positive likelihood ratio and negative likelihood ratio for D-dimer diagnosis of PJI were 3.14 (95% CI 1.98–4.96) and 0.26 (95% CI 0.16–0.41) (Fig. 7). According to previous studies, the incidence of PJI accounts for approximately 20% of arthroplasty revisions [25]. Therefore, 0.2 a priori probabilities were chosen, and the posterior probabilities were calculated by the likelihood ratio and the a priori probabilities. The posterior probability of PJI was 6%, indicating a negative D-dimer result.

### Univariate regression and subgroup analysis

**Fig. 8:**
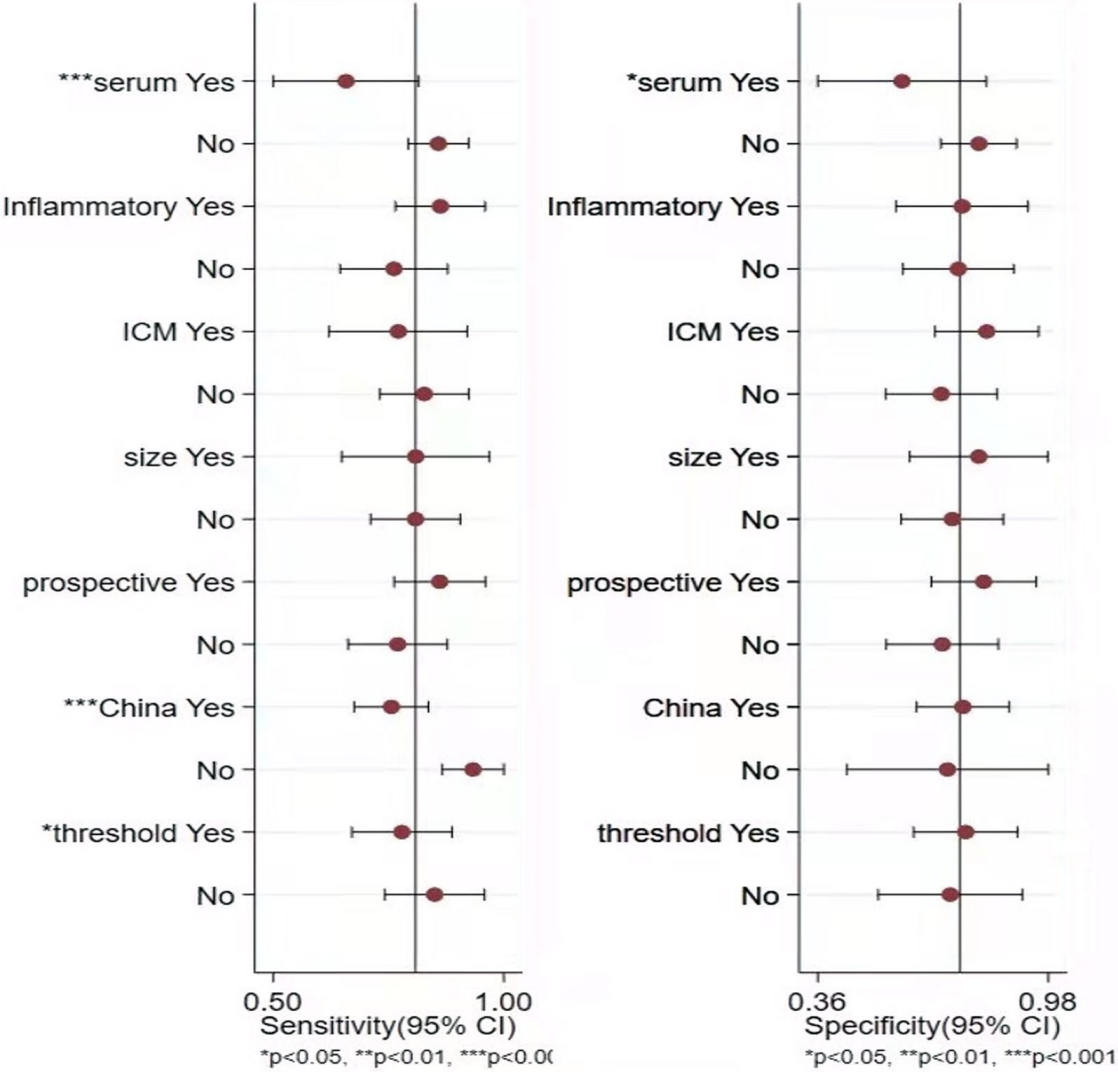
D-dimer diagnosis PJI regression analysis diagram

The heterogeneity of this study is evident from the forest plots. So we performed the following univariate regression and subgroup analysis (Fig. 8) to explore the sources of heterogeneity. Whether the threshold is the same, whether the sampling type and inflammatory diseases are excluded, the gold standard of diagnosis, whether the sample size is greater than 100, the type of study, the country of the source of the study, and the threshold. Univariate regression analysis showed that the type of sampling (serum or plasma) might be one of the main factors leading to heterogeneity. In addition, country and threshold are also possible factors leading to sensitivity heterogeneity. The results of the subgroup analysis showed a combined sensitivity and specificity of 0.85 (95% CI 0.74–0.95) and 0.72 (95% CI 0.52–0.91) for the four studies [12, 14, 19, 20] at 850 ng/ml (Table 3).

**Table 3:** Subgroup analysis for the diagnosis of PJI by D-dimer

Subgroup Analysis	Number of studies	Sensitivity (95% CI)	Specificity (95% CI)	Positive likelihood ratio (95% CI)	Negative likelihood ratio (95% CI)
All studies	10	0.81 [0.71, 0.88]	0.74 [0.61, 0.84]	3.1 [2.0, 5.0]	0.26 [0.16, 0.41]
Serum samples	7	0.86 [0.76, 0.92]	0.80 [0.65, 0.89]	4.2 [2.4, 7.3]	0.18 [0.11, 0.29]
China	8	0.76 [0.67, 0.83]	0.75 [0.65, 0.83]	3.0 [2.0, 4.5]	0.32 [0.22, 0.48]
Threshold value of 850 ng/ml	4	0.85 [0.69, 0.94]	0.72 [0.43, 0.90]	3.0 [1.3, 7.1]	0.21 [0.09, 0.45]
Threshold for other	6	0.78 [0.67, 0.86]	0.75 [0.63, 0.84]	3.2 [1.9, 5.2]	0.29 [0.17, 0.49]
Inflammatory disease exclusion	6	0.76 [0.65, 0.84]	0.74 [0.62, 0.83]	2.9 [1.9, 4.4]	0.33 [0.21, 0.50]
Inflammatory disease not ruled out	4	0.87 [0.71, 0.95]	0.75 [0.45, 0.92]	3.5 [1.3, 9.4]	0.18 [0.07, 0.42]
Sample size greater than 100	6	0.83 [0.68, 0.92]	0.71 [0.50, 0.85]	2.8 [1.5, 5.2]	0.24 [0.12, 0.49]
Sample size less than 100	4	0.78 [0.66, 0.86]	0.80 [0.71, 0.87]	3.9 [2.5, 6.2]	0.28 [0.17, 0.45]

### Publication bias risk

The Deeks funnel plot asymmetry test for D-dimers showed a tendency for both sides to be approximately symmetrical and *p* = 0.18, more significant than 0.05. Therefore, the publication bias was not statistically significant. The above tests confirmed the robustness of the results of our meta-analysis.

### Sensitivity analysis

To assess the credibility and consistency of the results, we omitted the included studies one by one for sensitivity analysis. Goodness-of-fit and bivariate normality analyses indicated moderate robustness of the binary model. Influence analysis and out detection identified only one outlier. We excluded this outlier and then performed the same analysis for the leaving study, and we found no significant change in the overall results.

### Additional meta-analysis

Reading again the 10 included studies, six of which analyzed the sensitivity and specificity of CRP and ESR for the diagnosis of PJI [12, 15, 19–21, 23], the sensitivity and specificity of CRP, ESR and D-dimer for the diagnosis of PJI were compared in these six studies, and it was found that CRP, ESR and D-dimer were more sensitive and specific for the diagnosis of PJI than CRP,ESR and D-dimer. There was no difference in sensitivity (*I*^2^ = 0, *P* = 0.777; *I*^2^ = 0, *P* = 0.798) or specificity (*I*^2^ = 0, *P* = 0.325; *I*^2^ = 0, *P* = 0.476) for the diagnosis of PJI; CRP and ESR, as the most commonly used serological indicators of infection, provide a good response to the overall infection status of the body, and D-dimer is similar to them in terms of diagnostic performance. This indicates that D-dimer has a high reference value as a serum biomarker in the diagnosis of PJI (Additional file 1).

## Discussion

This systematic review provides excellent diagnostic value evidence of an overall benefit of the D-dimer in diagnosing PJI. We suggest that D-dimer has high sensitivity and specificity in diagnosing periprosthetic infections, similar to CRP and ESR. Influence Analysis and Out Detection identified excluded low-quality trials and studies. We then performed the same analysis for the remaining studies, and we found no significant change in the overall results.

The diagnosis of PJI remains a challenge for orthopedic surgeons. First, the formation of pseudo-biofilm on the surface of the prosthesis in patients with PJI, which leads to sometimes negative results in pathogenic bacteria cultures. Secondly, when the chronic deep infection is present in PJI, it often resembles the clinical presentation of aseptic loosening of the prosthesis [26, 27]. With the development of research, more and more biological indicators for the diagnosis of PJI were identified, including synovial quantification-defensins, serological white blood cell count, ESR and CRP, interleukin-6, and Procalcitonin [28]. Several studies merit individual mention. Ribera et al.[10] believe that synovial D-dimer in foal infectious arthropathy is higher than the average level. Bytniewski et al.[29] showed that D-dimer levels change faster than ESR and CRP in patients with TKA in the early postoperative period and can rise rapidly and return to normal levels within a short period. Shahi et al.[12] proposed the threshold of D-dimer (850 ng/ml) for the first time and considered that serum D-dimer has high sensitivity (89%) and specificity (93%). Li et al.[13] speculated that D-dimer in patients with PJI may be a biomarker for late diagnosis. In 2018, Parvizi et al.[8] presented new evidence regarding the diagnosis of PJI based on evidence-based medicine that included D-dimer levels as a secondary criterion. Subsequently, an increasing number of studies have evaluated the role of D-dimer in the diagnosis of PJI by comparison with CRP and ESR, but different conclusions have been obtained. The sensitivity (64.5–95.9%) and specificity (32.3–92.75%) of D-dimer have varied considerably among studies.

In the univariate meta-regression, we concluded that differences in sampling type led to differences in insensitivity, which may account for the high heterogeneity. Monoclonal antibodies in serum, plasma, or whole blood are commonly used clinically to detect D-dimers, and four assays are generally used, whole blood agglutination assay, enzyme-linked immunosorbent assay, enzyme-linked immunofluorescence assay, and latex agglutination assay [30]. Of the 10 studies [12–15, 19–24] included, 3 studies [13, 19, 24] tested plasma D-dimer with a sensitivity of 0.66, whereas serum D-dimer showed a sensitivity of 0.86 in the other 7 studies [12, 14, 15, 20–23]. We hypothesize that the plasma and serum composition differences are the main reason for these results. The lack of fibrinogen and most of the depletion-induced coagulation factors in serum compared with plasma may contribute to the difference in D-dimer concentration between the two samples. However, to our knowledge, the evidence supporting our speculation is limited. A study by Korte and Riesen [31] found no difference in plasma and serum D-dimer concentrations. In contrast, Paniccia et al.[32] found higher serum D-dimer than plasma D-dimer in some pregnant women and healthy controls. Based on this meta-analysis, serum D-dimer was more sensitive than plasma D-dimer for diagnosing PJI. Still, the correlation between the two samples of D-dimer in patients with PJI needs to be further investigated.

The inflammatory-related diseases, although MSIS or ICM criteria have established index thresholds for diagnosing PJI, few studies have supported that these thresholds also apply to patients with PJI in inflammatory arthropathies [33]. Hence, the inclusion of systemic inflammatory diseases based on MSIS or ICM criteria may interfere with the diagnosis of PJI. For example, patients with rheumatoid arthritis (RA) have elevated D-dimer levels due to the degradation of large amounts of fibrin in the synovium [34]. In current study, we performed a subgroup analysis of inflammatory disease as an indicator of the impact of D-dimer in the diagnosis of periprosthetic infection, the results showed that inflammatory disease was not a source of article heterogeneity, which was inconsistent with the findings of Wang et al.[35]. Specific heterogeneity sources were mainly found in the countries studied (China and the United States). D-dimer levels have been reported to vary by race [36–38]. Therefore, we believe that the type of sampling and the country of study are the main factors affecting the diagnostic accuracy of D-dimer.

Ackmann et al. [39] found that Interleukin-6 (IL-6) combined with D-dimer was slightly more specific than CRP combined with D-dimer for the diagnosis of PJI, and both combinations were more specific than D-dimer alone for the diagnosis of PJI, but both were less sensitive than D-dimer (860 ng/ml). A study by Xu et al.[24] found that the sensitivity and specificity of D-dimer combined with ESR for the detection of PJI was lower than that of D-dimer combined with CRP, and the specificity of both groups for detecting PJI was higher than that of D-dimer alone for diagnosis, but the sensitivity of the combination was relatively lower. They suggest that the combination has a higher accuracy compared to a single indicator. Concurrent positive serum D-dimer and CRP can better exclude false positive tests for PJI. We therefore concluded that the combination of IL-6, CRP and ESR with D-dimer is promising in the diagnosis of PJI, and that IL-6 and CRP in particular deserve further investigation. However, AUC calculations for combined diagnosis were not performed in either study and the overall diagnostic value of the two groups could not be compared visually. Future related studies suggest the inclusion of a larger sample size and a more detailed comparison of different infection indicators combined with D-dimer and diagnosis alone in order to obtain more accurate test results.

### Study limitations and quality assessment

This meta-analysis has several advantages over previous similar meta-analyses. Firstly, the included literature is more significant, and the most recent relevant studies were included. Secondly, the reasons for heterogeneity were analyzed in more detail, including the type of blood sample, infection at other sites, diagnostic criteria, prosthesis type, study type, country, and threshold. Moreover, several limitations exist for the systematic review. Firstly, incomplete search and biased reporting. According to the search strategy and selection criteria, only 10 studies were included in this meta-analysis, among the included studies, four were prospective studies [12, 15, 19, 22], and only one was a multicenter study [13], which may be subject to publication bias and the unavailability of gray literature, which reduces the reliability of the study. Secondly, the age, sex ratio, number, different affected joints, follow-up time, and prosthesis materials were not fully counted in this study, and possible bias in the results may exist. The design of future clinical trials could be improved in the future to address these issues. Finally, there is no gold standard for PJI detection, and the standard gold test used in this study was only approximate. Some positive patients were still missed because the gold standard was not detectable. There is also some measurement bias due to differences in sampling types and thresholds. Therefore, further extensive prospective multicenter randomized controlled studies are needed to confirm this conclusion. This study included 9 papers with a low risk of bias and 1 article with a high risk of bias, which is somewhat representative of the current state of clinical research.

## Conclusion

The D-dimer has an excellent diagnostic value for PJI, but the specificity is limited. Therefore, we can conclude that D-dimer is a promising serological biomarker for diagnosing PJI and can be used in combination with other biomarkers or as an adjunct to other diagnostic tools to improve the diagnostic performance.

## Supplementary Information

**Additional file 1**. Additional meta-analysis.

## Data Availability

All data generated or used during the study appear in the submitted article.

## Abbreviations

PJI: Periprosthetic joint infection;
THA: Total hip arthroplasty
TKA: Total knee arthroplasty
CI: Confidence interval
MSIS: Musculoskeletal Infection Society
ICM: International Consensus Meeting
CRP: C-reactive proteinand
ESR: Erythrocyte sedimentation rate
IL-6: Interleukin-6
TP: True positive
FP: False positive
TN: True negative
FN: False negative
AUC: Area under the curve
SROC: Summary receiver operator characteristic curve
