# Supplementary material for: The accuracy of D-dimer in the diagnosis of periprosthetic infections: a systematic review and meta-analysis
Source: J Orthop Surg Res. 2022 Feb 16;17:99. doi: 10.1186/s13018-022-03001-y (PMC8848660; doi:10.1186/s13018-022-03001-y)
Supplement: Supplementary file 1 — Additional file 1. Additional meta-analysis. [file 13018_2022_3001_MOESM1_ESM.docx]

Additional meta-analysis data：


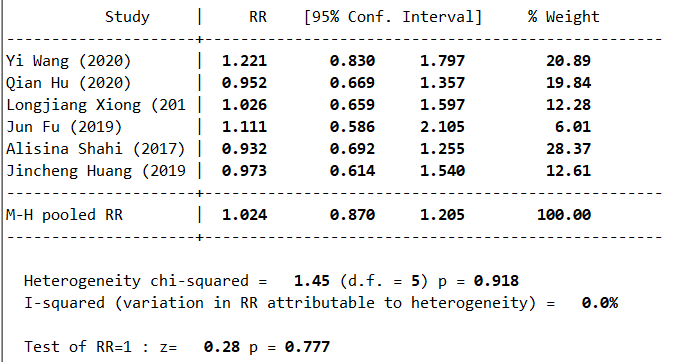


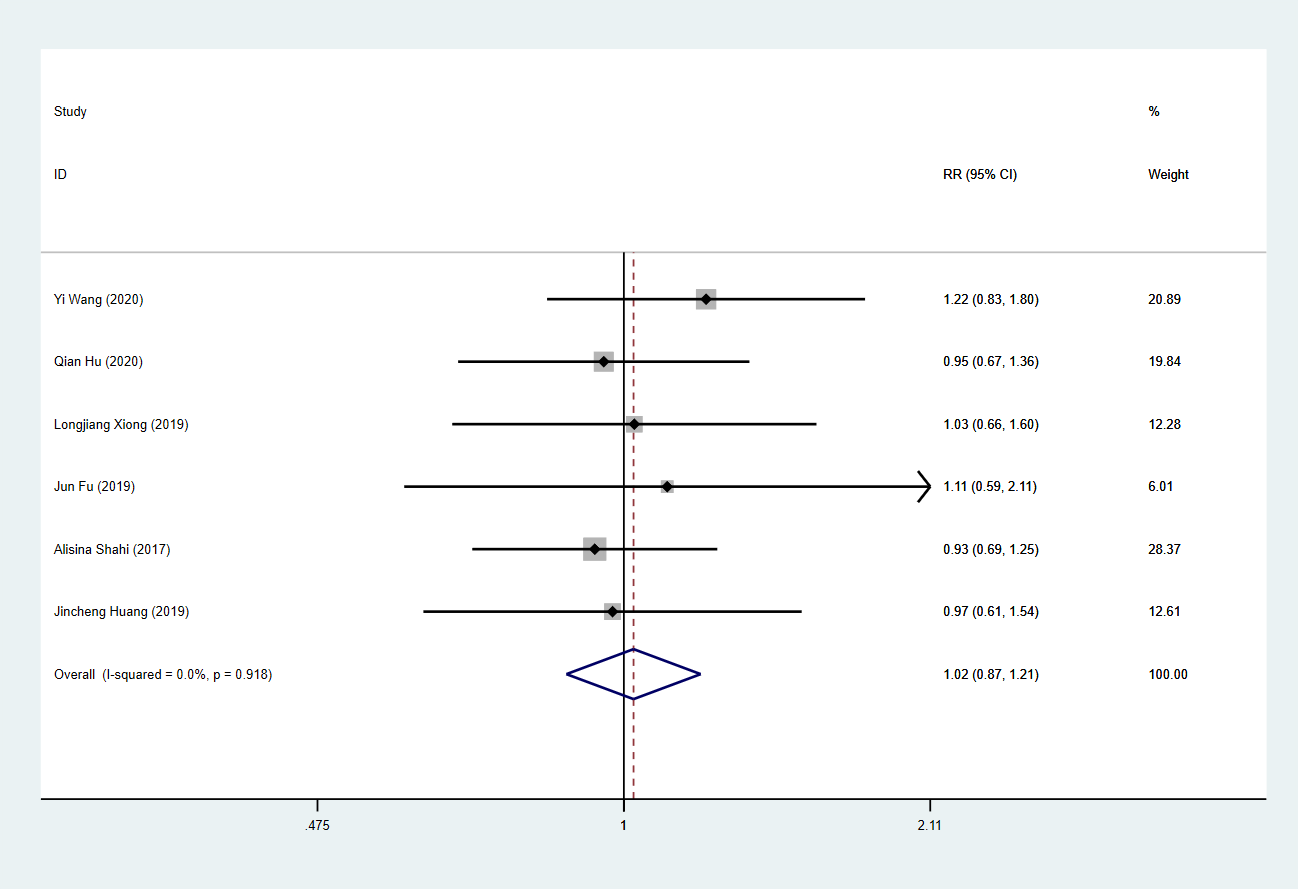


A meta-analysis comparing the sensitivity of CRP and D-dimer in the diagnosis of PJI


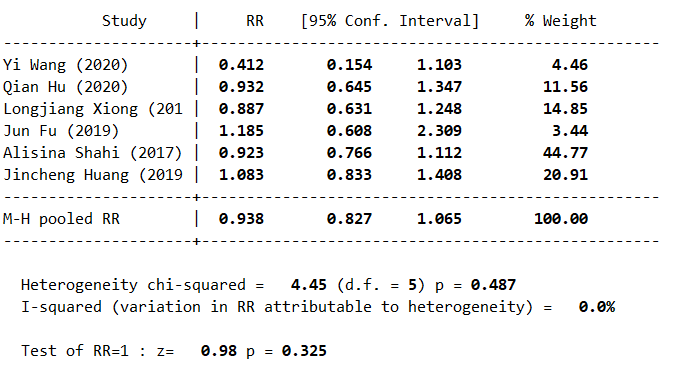


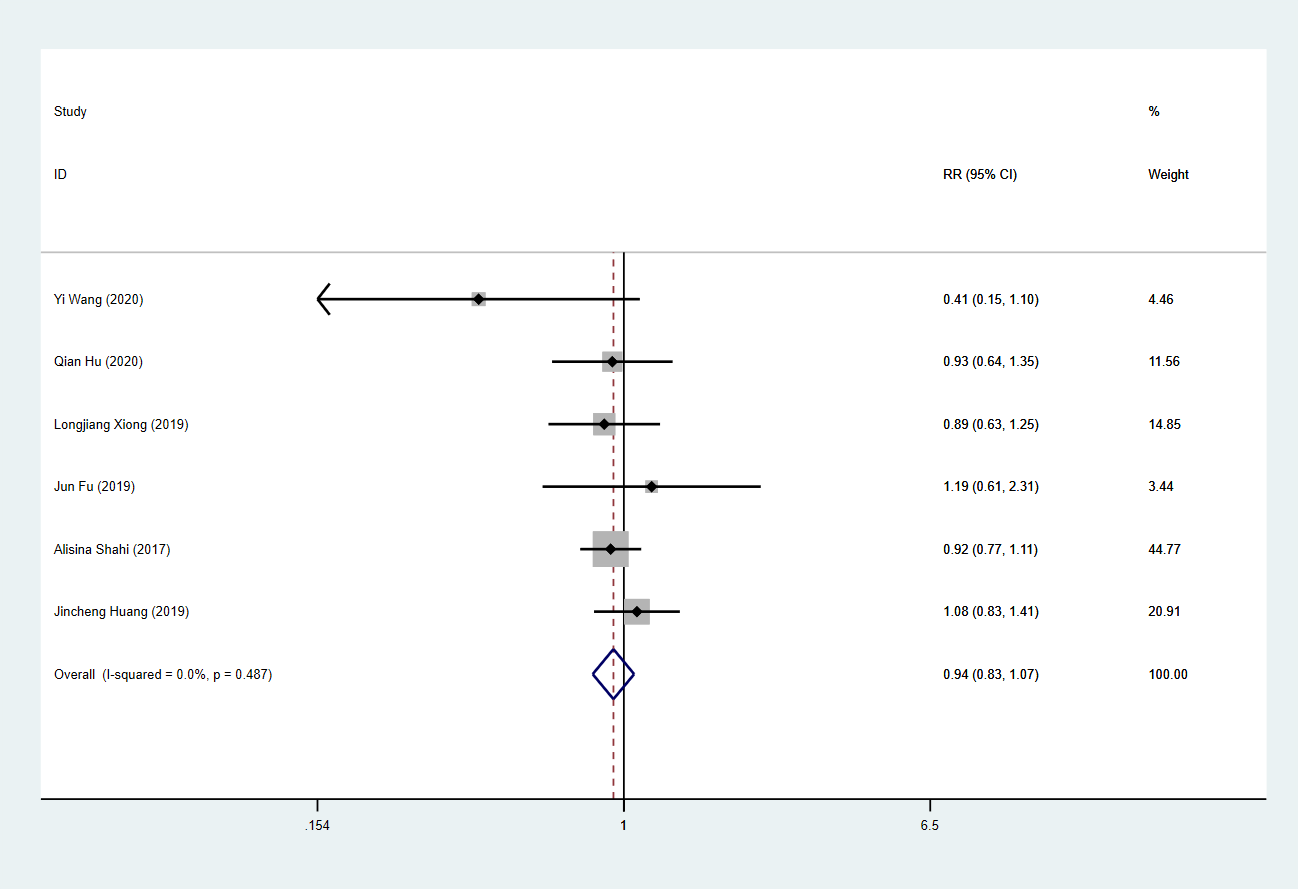


A meta-analysis comparing the specificity of CRP and D-dimer in the diagnosis of PJI


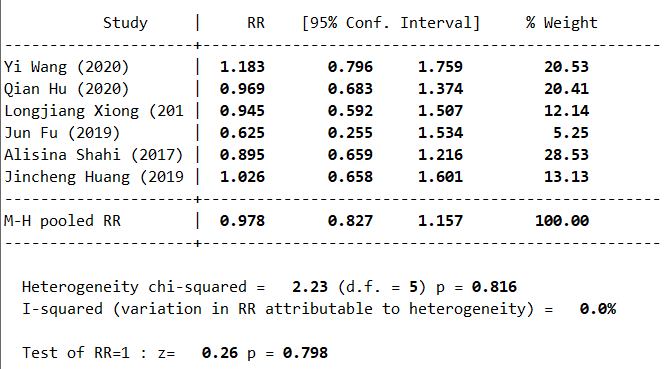


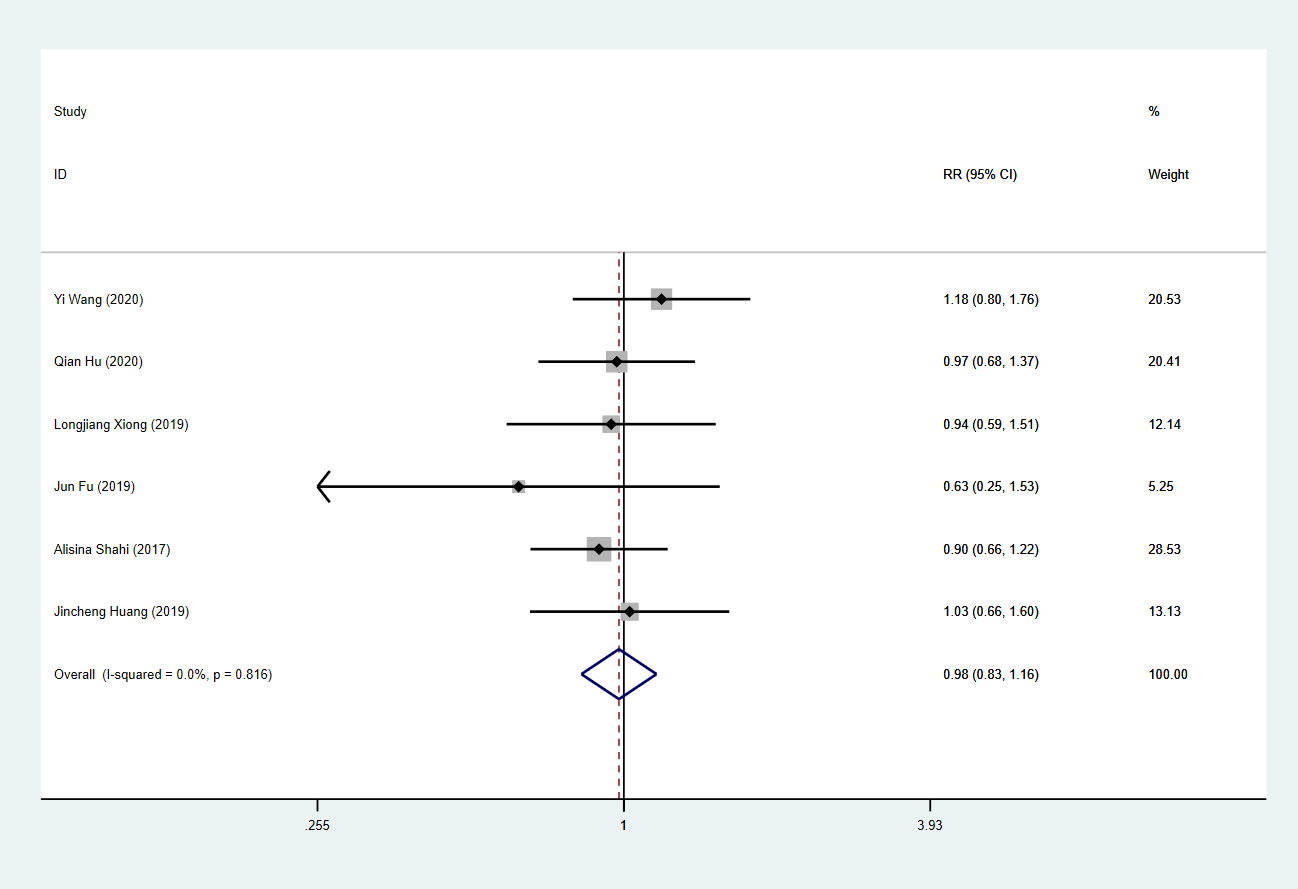


A meta-analysis comparing the sensitivity of ESR and D-dimer in the diagnosis of PJI


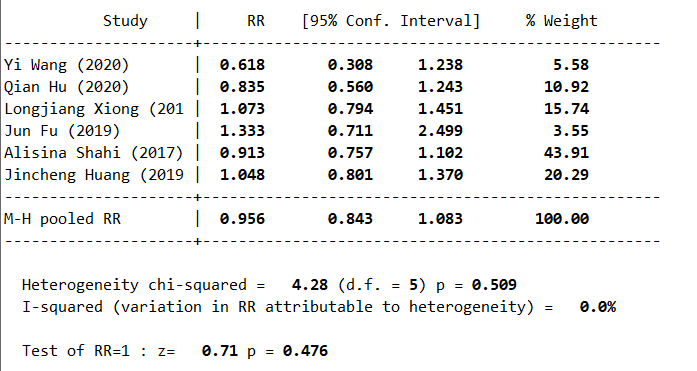


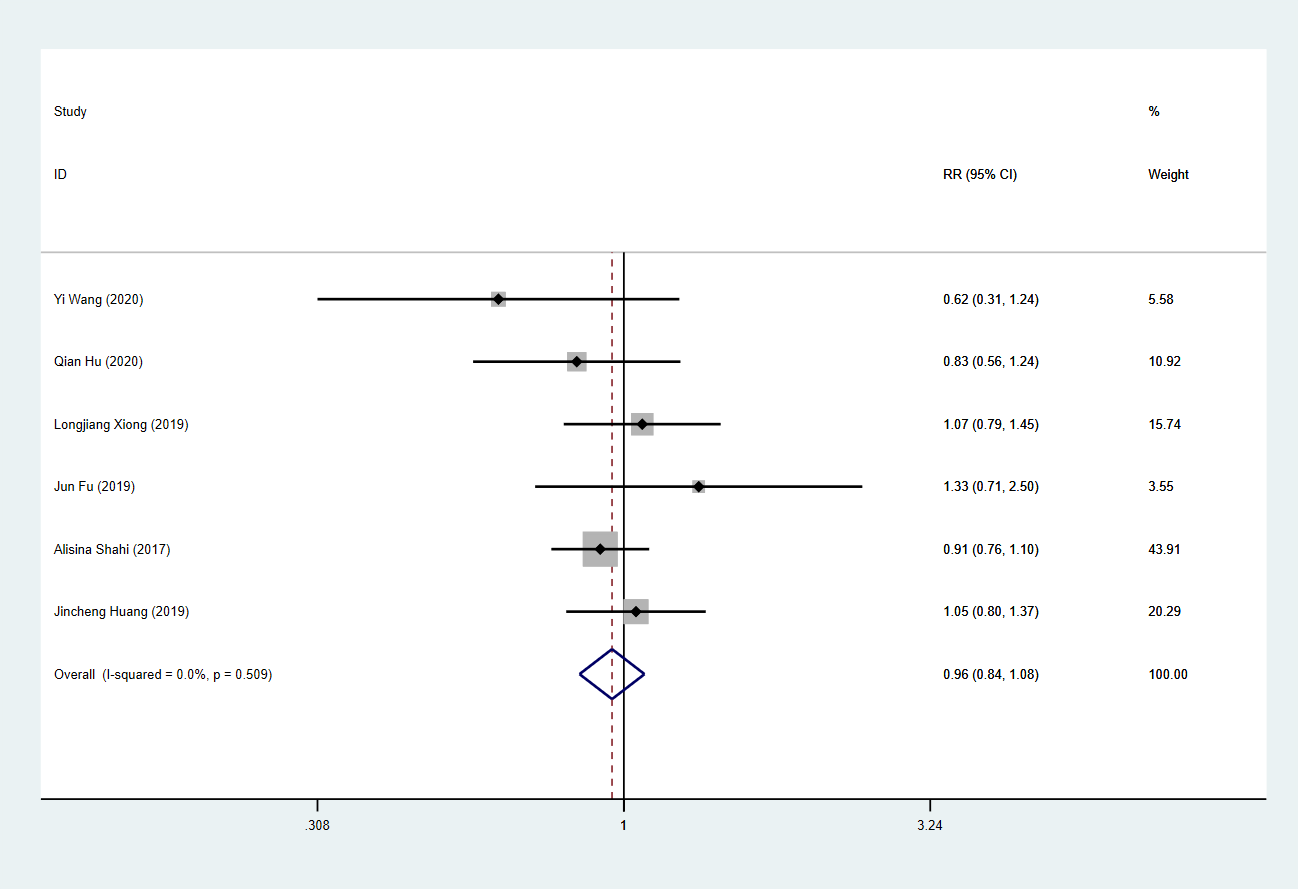


A meta-analysis comparing the specificity of ESR and D-dimer in the diagnosis of PJI
